# Longitudinal neurofunctional changes in medication overuse headache patients after mindfulness practice in a randomized controlled trial (the MIND-CM study)

**DOI:** 10.1186/s10194-024-01803-5

**Published:** 2024-06-11

**Authors:** Davide Fedeli, Giuseppe Ciullo, Greta Demichelis, Jean Paul Medina Carrion, Maria Grazia Bruzzone, Emilio Ciusani, Alessandra Erbetta, Stefania Ferraro, Marina Grisoli, Erika Guastafierro, Domenico D’Amico, Alberto Raggi, Anna Nigri, Licia Grazzi

**Affiliations:** 1 Department of Neuroradiology, Fondazione IRCCS Istituto Neurologico Carlo Besta, Via Celoria 11, Milano, Italy; 2 Department of Medicine and Surgery, University of Parma, Via Volturno 39, Parma, 43125 Italy; 3 Department of Diagnostic and Technology, Fondazione IRCCS Istituto Neurologico Carlo Besta, Milano, Italy; 4 School of Life Science and Technology, MOE Key Laboratory for Neuroinformation, University of Electronic Science and Technology of China, Chengdu, China; 5 Neurology, Public Health and Disability Unit, Fondazione IRCCS Istituto Neurologico Carlo Besta, Milano, Italy; 6 Neuroalgology Unit and Headache Center, Fondazione IRCCS Istituto Neurologico Carlo Besta, Milano, Italy

**Keywords:** Mindfulness, Medication overuse headache, Resting state fMRI, Salience network, Chronic pain, Cortical thickness, Functional connectivity, Headache, Migraine, Pain management

## Abstract

**Background:**

Mindfulness practice has gained interest in the management of Chronic Migraine associated with Medication Overuse Headache (CM-MOH). Mindfulness is characterized by present-moment self-awareness and relies on attention control and emotion regulation, improving headache-related pain management. Mindfulness modulates the Default Mode Network (DMN), Salience Network (SN), and Fronto-Parietal Network (FPN) functional connectivity. However, the neural mechanisms underlying headache-related pain management with mindfulness are still unclear. In this study, we tested neurofunctional changes after mindfulness practice added to pharmacological treatment as usual in CM-MOH patients.

**Methods:**

The present study is a longitudinal phase-III single-blind Randomized Controlled Trial (MIND-CM study; NCT03671681). Patients had a diagnosis of CM-MOH, no history of neurological and severe psychiatric comorbidities, and were attending our specialty headache centre. Patients were divided in Treatment as Usual (TaU) and mindfulness added to TaU (TaU + MIND) groups. Patients underwent a neuroimaging and clinical assessment before the treatment and after one year. Longitudinal comparisons of DMN, SN, and FPN connectivity were performed between groups and correlated with clinical changes. Vertex-wise analysis was performed to assess cortical thickness changes.

**Results:**

177 CM-MOH patients were randomized to either TaU group or TaU + MIND group. Thirty-four patients, divided in 17 TaU and 17 TaU + MIND, completed the neuroimaging follow-up. At the follow-up, both groups showed an improvement in most clinical variables, whereas only TaU + MIND patients showed a significant headache frequency reduction (*p* = 0.028). After one year, TaU + MIND patients showed greater SN functional connectivity with the left posterior insula (p-FWE = 0.007) and sensorimotor cortex (p-FWE = 0.026). In TaU + MIND patients only, greater SN-insular connectivity was associated with improved depression scores (*r* = -0.51, *p* = 0.038). A longitudinal increase in cortical thickness was observed in the insular cluster in these patients (*p* = 0.015). Increased anterior cingulate cortex thickness was also reported in TaU + MIND group (p-FWE = 0.02).

**Conclusions:**

Increased SN-insular connectivity might modulate chronic pain perception and the management of negative emotions. Enhanced SN-sensorimotor connectivity could reflect improved body-awareness of painful sensations. Expanded cingulate cortex thickness might sustain improved cognitive processing of nociceptive information. Our findings unveil the therapeutic potential of mindfulness and the underlying neural mechanisms in CM-MOH patients.

**Trial Registration:**

Name of Registry; MIND-CM study; Registration Number ClinicalTrials.gov identifier: NCT0367168; Registration Date: 14/09/2018

## Background

Chronic Migraine associated with Medication Overuse Headache (CM-MOH) is a painful and debilitating neurological disorder affecting patients with a pre-existing primary headache. It is defined as a secondary headache occurring 15 or more days per month due to the overuse of acute medications for at least three months [1, 2]. Paradoxically, medication overuse in these patients can worsen headache frequency, pain intensity, and medication addiction, exacerbating the overall burden of the condition [3]. On this basis, there is growing interest in adopting non-pharmacological approaches, either alone or to potentiate preventive treatments of CM-MOH patients [4, 5]. Mindfulness-based interventions have gained attention as a potential adjunctive therapy for migraine pain management [4–7]. Mindfulness is a clinical practice rooted in the meditation tradition that cultivates present-moment awareness and non-judgmental acceptance [8]. Most recent investigations have specifically revealed the beneficial effects of mindfulness practice in CM-MOH patients [9–11]. In particular, Grazzi and colleagues [11]showed that mindfulness added to treatment as usual (TaU + MIND) in these patients is more effective than TaU alone in reducing headache frequency and medication intake, mitigating pain-related costs and interference with daily functioning, thus improving the overall quality of life. Nevertheless, the neural processes underlying these effects remain largely elusive.

Only recently, neuroimaging studies have begun to shed light on the neurofunctional mechanisms implicated in the therapeutic effects of mindfulness in pain perception and modulation [12–18]. Notably, mindfulness practice has been suggested to influence the functional connectivity of three interacting brain networks, the Default Mode Network (DMN), the Salience Network (SN), and the Fronto-Parietal Network (FPN) [14, 16]. This “triple network model*”* [19], is responsible for switching attentional focus between salient stimuli and alternate states of spontaneous and deliberate thought [19–21]. Mindfulness-related areas overlap to a broad extent with brain regions involved in pain processing and modulation^18^ and part of the SN. Moreover, CM-MOH has been linked to abnormal functional activity in brain regions involved in pain processing, which substantially overlap with the nodes of the SN [22–26].

On this basis, we suggest that mindfulness practice in CM-MOH patients could be specifically effective in modulating the SN connectivity amongst the networks involved in the triple network model.

To deepen the knowledge of the neural mechanisms that underlie the known therapeutic effects of Mindfulness-based interventions in CM-MOH patients, we performed the MIND-CM study, a Randomized Controlled Trial that demonstrated the superiority of adding on a mindfulness-based treatment (TaU + MIND) to TaU alone [11] (NCT03671681). As part of this Randomized Controlled Trial, a portion of patients underwent a longitudinal neuroimaging study that compared the longitudinal resting-state fMRI (rs-fMRI) neurofunctional changes occurring in the DMN, SN, and FPN networks between TaU + MIND and TaU patients. Moreover, the study aims to assess if the observed longitudinal functional connectivity alterations were accompanied by co-occurring clinical and cortical thickness changes. Based on previous evidence [12, 15, 18], we expect TaU + MIND patients to show increased connectivity and changes in cortical thickness in networks associated with the triple network model as well as areas of pain processing and perception. We also expect TaU + MIND group to show better clinical outcomes after one year correlated with longitudinal neurofunctional changes [11, 18].

## Methods

### Trial design and participants

MIND-CM study is a phase-III single-blind Randomized Controlled Trial including CM-MOH patients attending our specialty headache centre for a structured withdrawal treatment. The study was reported in accordance with the CONSORT guidelines [27], and registered on clinicaltrials.gov (NCT03671681). The study started in November 2018; the last patient was enrolled in December 2021; the last follow-up was completed in November 2022.

Inclusion criteria consisted of diagnosis of CM-MOH (codes 1.3 and 8.2) according to the Headache Classification Committee of the International Headache Society guidelines, 3rd edition [2]. Patients with neurological and severe psychiatric comorbidities, pregnancy, other-than-MOH secondary headaches, withdrawal from Medication Overuse Headache twice or more in the last two years, and any previous experience with mindfulness practice were excluded.

All participants gave their written consent. The study was approved by the Ethical Committee of the Fondazione IRCCS Istituto Neurologico “Carlo Besta”, Milan (approval no. 51/2018).

### Interventions

The detailed description of the clinical protocol is reported in Grazzi et al. [11].

#### TaU protocol

The TaU protocol involved withdrawing overused symptomatic medications, tailored prophylaxis, and education on how to correctly use acute medications and how to maintain a healthy lifestyle (e.g., adequate sleep/wake patterns, healthy eating and hydration, physical activity, avoidance of headache triggers) [28]. Prescribed preventive compounds were primarily either neuromodulators (e.g., topiramate or valproate) or antidepressants (e.g., tricyclics or SSRIs). Based on each patient’s clinical profile assessment, other compounds were prescribed when necessary (e.g., beta-blockers).

#### TaU + MIND protocol

TaU + MIND protocol included TaU with the add-on of six guided sessions of Mindfulness-based intervention. During mindfulness practice, patients focused on the present moment, concentrated on their breath, scanned their body, and paid attention to their bodily sensations [11]. The TaU + MIND program consisted of six 90-minute-long weekly sessions administrated by an expert trained neurologist (L.G.). Within each session, the duration of the mindfulness practice progressively increased from 5 min in the first session to 25 min at the end of the intervention. From the third session onwards, patients were also familiarized with practicing at home with a short session that focused just on breathing. Moreover, patients were instructed to regularly perform 7–10 min of daily home self-practice guided by a 12-minute audio file.

#### Clinical and neuroimaging assessment

Patients underwent a clinical evaluation at two timepoints: a baseline visit at enrolment (T0) and a follow-up visit after 12 months (T1).

In the clinical assessment, the frequency of headache attacks and symptomatic medications intake (triptan and non-steroidal anti-inflammatory drugs) in the last three months were collected. Moreover, the following tests were administered: Beck Depression Inventory-II (BDI) for depression symptoms [29], State-Trait Anxiety Inventory (STAI-Y) for state and trait anxiety [30], Allodynia Symptoms Checklist (ASC-12) for cutaneous allodynia [31], and Mindful Attention and Awareness Scale (MAAS) for self-awareness [32].

An MRI scan was performed both at T0 and at T1.

### Outcomes

The primary outcome of the MIND-CM study consisted of 50% or more headache frequency reduction, in the last 3 months, assessed at 12 months and was previously reported by Grazzi et al. [11] along with other secondary outcomes, as reported in the registered Randomized Controlled Trial protocol.

The registered MRI outcome consists of changes in neuroimaging patterns specific to TaU + MIND patients treated with mindfulness added to prescribed therapy with neuromodulators or antidepressants with respect to the TaU group. Unfortunately, the COVID-19 pandemic interfered with our Randomized Controlled Trial, therefore several patients could not perform the follow-up assessment at our institute due to mobility restrictions. Hence, it was not possible to test the effects associated with prophylaxis (i.e., neuromodulators vs. antidepressants) on functional connectivity amongst TaU + MIND and TaU patients. Therefore, the expected outcome of our study is a change in the functional connectivity of TaU + MIND patients irrespective of the pharmacological prophylaxis.

### Sample size calculation

Based on sample size computation, randomization was estimated to involve 170 patients [11]. This calculation was based on the hypothesis that 48% of TaU patients would achieve ≥ 50% headache-day reduction after one year, and that mindfulness practice add-on would increase this figure by 20%. At least 75 patients per group (TaU + MIND and TaU) were considered necessary with alpha set at 0.05 and power at 80%, with an estimate of 12% drop-out at follow-up.

### Randomization

Patients were randomly assigned to TaU and TaU + MIND in a 1:1 ratio, using a computer-generated list (simple randomization). The enrolling and evaluating neurologist (D.D.) remained blind to the allocation; A.R. prepared the randomization list and a set of opaque envelopes, randomized patients and handled data collection with other researchers; mindfulness sessions were conducted by L.G.

After screening, eligible patients voluntarily joined the study by signing the informed consent, ensuring they understood the purpose and the blinded evaluation by the neurologist (D.D.). Patients were reminded not to disclose their group allocation. A subsample of ~ 60 patients was expected to undergo MRI acquisition both at the baseline (T0) and at the follow-up (T1), in order to split the sample into 15 TaU + MIND patients treated with antidepressants, 15 TaU + MIND patients treated with neuromodulators, 15 TaU patients treated with antidepressants, and 15 TaU patients treated with neuromodulators. No sample size calculation was performed at this stage to determine the number of participants necessary for MRI sessions.

### MRI acquisition

MRI acquisitions were performed at the Department of Neuroradiology, Fondazione IRCCS Istituto Neurologico “Carlo Besta”, Milan (Italy), on a 3T scanner (Achieva dStream, Philips Healthcare BV, Best, NL) using a 32-channel head coil. All MRI images were visually inspected by an experienced neuroradiologist (A.E.).

At each timepoint (T0 and T1), a functional rs-fMRI sequence (eyes opened; T2*-weighted BOLD echo-planar imaging gradient-echo sequence; Repetition Time [TR] = 2000ms; Echo Time [TE] = 30ms; Field-of-View [FOV] = 80 × 80mm; voxel size = 3 × 3 × 3.2mm^3^; interslice gap = 0.4 mm; flip angle = 80°; 34 axial slices; 450 volumes; Phase Encoding direction = posterior/anterior; acquisition duration = 15 min), a high-resolution structural 3D T1-weighted image (TR = 8.11s; TE = 3.71ms; FOV = 240 × 240mm; voxel size = 1 × 1 × 1mm^3^; flip angle = 8°; 185 sagittal slices), and a 3D FLAIR sequence (TR = 5000ms; TE = 30ms; inversion time = 1700ms; FOV = 240 × 240mm; voxel size = 1 × 1 × 1mm^3^; flip angle = 90°; 180 axial slices) were acquired.

The quality of structural and functional images was assessed and visually inspected using the BIDS-compliant software MRIQC [33].

### MRI pre-processing

Connectivity analyses were performed using CONN connectivity toolbox (v. 20.b; running in Matlab 2020b) [34] with the “default_MNI” preprocessing and denoising pipeline (smoothing = 6 mm^3^, aCompCor denoising, 0.008–0.1 Hz bandpass filter). T1-weighted and FLAIR images were bias field corrected and cortical surfaces were reconstructed with the “recon-all” pipeline as implemented in FreeSurfer (version 7; https://surfer.nmr.mgh.harvard.edu/); smoothing = 15 mm^3^. FreeSurfer longitudinal pipeline was run for each patient [35].

### Statistical analyses

Statistical analyses were conducted to: (i) assess differences in demographic and clinical variables between groups at T0; (ii) investigate treatment-related longitudinal differences in clinical variables between groups; (iii) assess distinct longitudinal changes of SN, DMN, and FPN seed-based resting state functional connectivity between groups; (iv) estimate correlations between connectivity changes and improvements in clinical variables; and (v) assess longitudinal structural whole-brain changes between groups.

#### Demographic and clinical data analyses

Normality test was performed for all variables using Shapiro-Wilk test. The following variables were assessed only at T0 and compared between groups (i.e., TaU + MIND and TaU) using the U Mann-Whitney or the χ^2^ test: age (in years), sex assigned at birth, years of formal education, years of migraine duration (i.e., age at T0 minus reported age at migraine onset), years of chronic migraine duration, type of overused medications, and prophylaxis assigned as treatment.

To determine differences between groups in clinical variables at T0 (frequency of headache attacks, medication intake, depression severity, state and trait anxiety, allodynia, and self-awareness score), the U Mann-Whitney test was used.

Moreover, longitudinal differences in clinical variables within groups (TaU + MIND T1 vs. T0; TaU T1 vs. T0) were assessed using Wilcoxon signed-rank test.

Finally, to investigate treatment-related longitudinal differences (Δ = T1 – T0) in clinical variables between groups, changes between T1 and T0 (Δ TaU + MIND vs. Δ TaU) in the same clinical variables were assessed using U Mann-Whitney test.

All between-group comparisons were performed and reported both with and without whole-sample outlier removal using each variable’s mean ± 3 standard deviations as a threshold. A *p* < 0.05 was considered statistically significant. All analyses were performed with R 4.2.1.

#### Resting-state fMRI analyses

For each participant at each timepoint, a seed-to-voxel approach was used to estimate the whole-brain connectivity of DMN (medial prefrontal cortex, bilateral lateral parietal region, posterior cingulate cortex), SN (anterior cingulate cortex, bilateral anterior insula, bilateral rostral prefrontal cortex, bilateral supramarginal gyrus), and FPN (bilateral lateral prefrontal cortex, bilateral posterior parietal cortex) using the available pre-defined regions in the networks atlas as implemented in CONN [34]. For each rs-fMRI network, Pearson’s correlation coefficients were computed between the average of signal time-series of each region of interest and all brain voxels. Correlation coefficients were then converted to Z scores with the R-to-Z Fisher transform. Connectivity maps of each network (DMN, SN, and FPN) were entered into a separate second-level general linear model to assess functional connectivity differences in network profiles between TaU + MIND and TaU patients. Between-group functional connectivity differences were assessed at the baseline (TaU + MIND T0 vs. TaU T0) and at the follow-up (Δ TaU + MIND vs. Δ TaU). Age and sex assigned at birth were included as nuisance covariates. All results were FWE corrected at *p* < 0.05 at the cluster level and *p* < 0.001 uncorrected at the voxel level (i.e., a *p* < 0.001 cluster-defining primary threshold was applied to all voxels, then FWE correction was applied to the resulting clusters to correct p-values for multiple comparisons to avoid false-positive findings). Finally, for all significant results, effect size (Cohen’s *d*) was estimated. A Cohen’s *d* > 0.5 was considered as indicative of a large effect.

#### Correlational analyses

To estimate correlations between functional connectivity changes and improvements in clinical variables, connectivity values from significant clusters resulting from rs-fMRI longitudinal between-group comparisons (Δ TaU + MIND vs. Δ TaU) were extracted. Normality of variables was tested with Shapiro-Wilk test. Then, connectivity values were correlated to corresponding variations in clinical variables (Δ) using Pearson’s correlations (*r*) separately for each group.

#### Structural analyses

Two separate structural analyses were performed to assess whether the functional connectivity changes were accompanied by co-occurring morphological variations: a region of interest-based analysis and a whole-brain analysis.

For statistically significant functional connectivity clusters obtained in rs-fMRI longitudinal between-group comparisons (Δ TaU + MIND vs. Δ TaU), a region of interest-based analysis was applied. Mean cortical thickness values were extracted from significant functional clusters with FreeSurfer in each patient at each timepoint. Then, longitudinal between-group comparisons were performed using U-Mann-Whitney test (Δ TaU + MIND vs. Δ TaU).

To evaluate structural alterations across the entire brain between the TaU + MIND and TaU groups at baseline and longitudinally, a whole-brain analysis was performed. A general linear model was fitted using FreeSurfer vertex-wise analyses to ensure that no between-group differences in whole brain cortical thickness were present at T0 (TaU + MIND T0 vs. TaU T0). Finally, FreeSurfer longitudinal pipeline was adopted to compare vertex-wise longitudinal changes between TaU + MIND and TaU groups (Δ TaU + MIND vs. Δ TaU). In vertex-wise analysis, age was considered as a covariate. All results were corrected for multiple comparisons and considered significant if surviving a *p* < 0.001 vertex-wise threshold and a p-FWE < 0.05 cluster-wise threshold. For all significant results, effect size (Cohen’s *d*) was estimated.

## Results

### Participants

A total of 191 CM-MOH patients were invited to participate in the clinical trial. Fourteen patients were excluded from the trial since they did not meet the inclusion criteria. A total of 177 patients were randomized in two groups: 89 TaU, and 88 TaU + MIND. MRI acquisition at T0 was performed for a subsample of 42 TaU patients and 49 TaU + MIND patients.

**Fig. 1:**
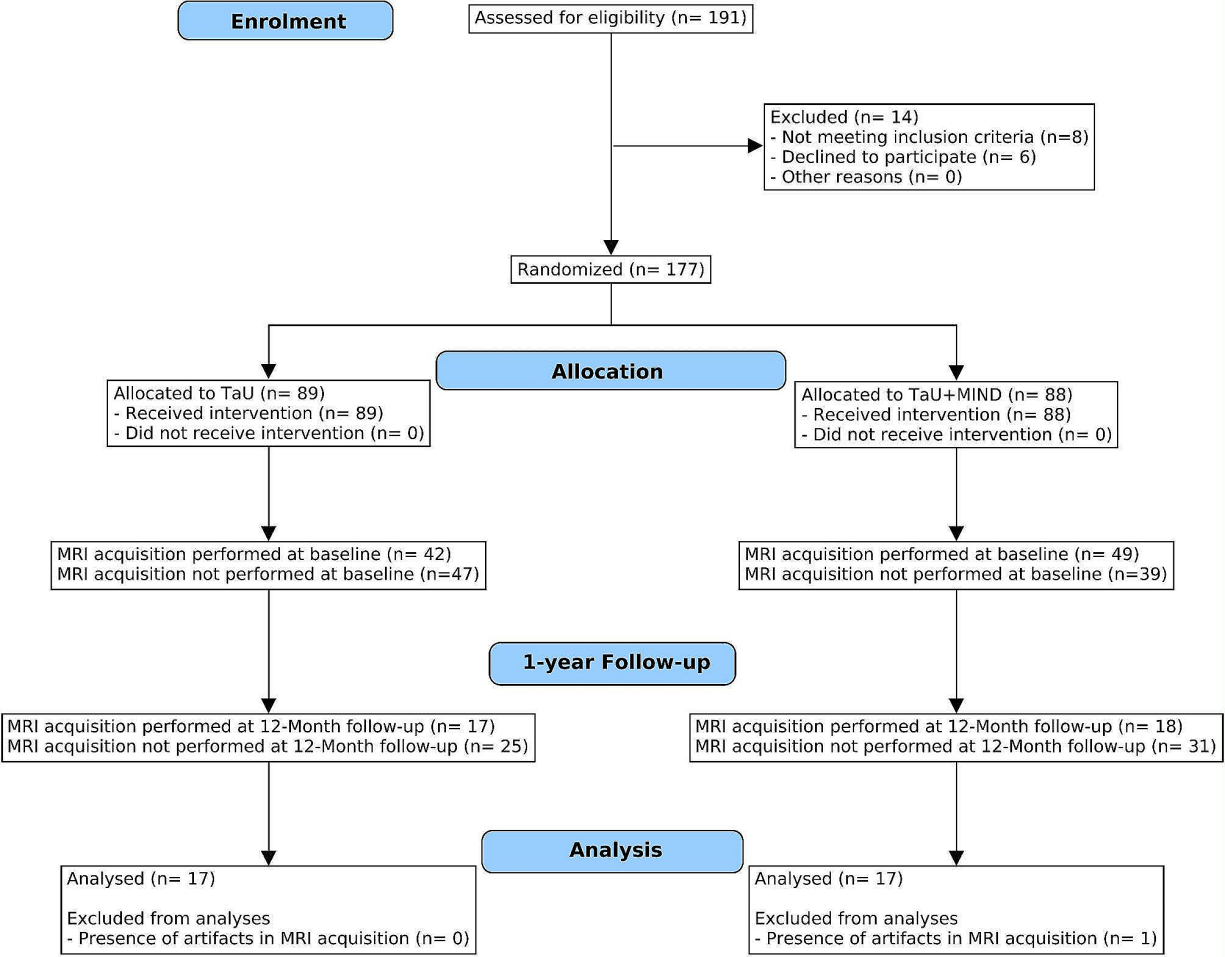
CONSORT flow diagram. Trial profile of 191 patients screened for eligibility. 91 patients were included in the MRI subsample. A total of 35 participants completed the study, of whom 34 analysed

Due to COVID-19 pandemics, and the important mobility restrictions connected to it, several patients could not attend the T1 evaluation (which started on January 2020). At the end of the trial, we were able to include 35 patients in the longitudinal analyses. One additional patient was removed from the analyses because of the presence of artifacts in the resting state fMRI sequence. Hence, the final sample consisted of thirty-four (*N* = 34) CM-MOH patients: 17 TaU and 17 TaU + MIND. The last follow-up was completed in November 2022. A CONSORT flow diagram is reported in Fig. 1.

TaU and TaU + MIND groups were largely comparable for age, sex assigned at birth, education, migraine duration, chronic migraine duration, type of overused medications (i, e., non-steroidal anti-inflammatory drugs, triptans, both non-steroidal anti-inflammatory drugs and triptans), and prophylaxis (i.e., antidepressants, neuromodulators) (Table 1). Aura symptoms were not reported before or after any MRI acquisition or in patients’ clinical history. No adverse effects of interventions were reported by the patients. Patients did not report headache before or during MRI acquisition. All patients were in interictal migraine phase at the time of MRI session.

**Table 1:** Data at the baseline (T0) for TaU + MIND and TaU patients

		TaU + MIND(*N* = 17)	TaU(*N* = 17)	U	χ2	*p*-value
Age (years)	Median (Q1, Q3)	50 (44, 58)	50 (36, 55)	176.5	--	0.277
Sex assigned at birth	Males, Females	2, 15	2, 15	--	0	1.000
Education Level (years)	Median (Q1, Q3)	13 (12, 13)	13 (13, 17)	98	--	0.090
Migraine Duration (years)	Median (Q1, Q3)	30 (14, 40)	26 (18, 43)	141	--	0.919
Chronic Migraine Duration (years)	Median (Q1, Q3)	15 (6, 22)	12 (3, 22)	163	--	0.535
Overused Medications	NSAIDs, Triptans, Polyabuse	9, 5, 3	6, 8, 3	--	1.292	0.524
Prophylaxis	Antidepressant, Neuromodulator	5, 12	5, 12	--	0	1.000

The results of U Mann-Whitney and χ^2^ tests are reported. *Abbreviations* TaU + MIND = Mindfulness added to Treatment as Usual group; TaU = Treatment as Usual group; Q1 = first quartile; Q3 = third quartile; NSAIDs = non-steroidal anti-inflammatory drugs; Polyabuse = abuse of both NSAIDs and Triptans

### Clinical data analyses

No significant differences were found between TaU + MIND and TaU patients at T0 for demographic and clinical variables (Tables 1 and 2). In general, both groups improved in clinical profile at T1 (TaU + MIND T1 vs. T0; TaU T1 vs. T0) (Table 2). Longitudinal between-group comparisons in clinical changes (Δ TaU + MIND vs. Δ TaU) showed a tendency towards a greater improvement in TaU + MIND patients compared to TaU patients, especially for the frequency of headache attacks which was almost significant (*p* = 0.07). This latter finding became significant after mean ± 3 standard deviations outlier removal (*p* = 0.028, one outlier) (Table 2).

**Table 2:** Clinical data at the baseline (T0) and at follow-up (T1) for TaU + MIND and TaU patients

	Tau + MIND		TaU		TaU + MIND T0 vs. TaU T0	TaU + MIND T1 vs. T0	TaU T1 vs. T0	Δ TaU + MIND vs. Δ TaU
	T0	T1	T0	T1				
	Median (Q1,Q3)	Median (Q1,Q3)	Median (Q1,Q3)	Median (Q1,Q3)	U; *p*-value	U; *p*-value	U; *p*-value	U; *p*-value
Frequency of headache attacks	60 (50, 70)	23 (15, 25)	56 (45, 68)	32 (20, 45)	156.5; 0.689	**141; 0.002**	**134; <0.001**	91.5; 0.070 ^c^
Medication intake	87 (54, 126)	23 (9, 36)	93 (75, 156)	36 (9, 72)	130; 0.629 ^a^	**153; <0.001**	**131; 0.001**	119; 0.389 ^d^
STAI-Y1	53 (42, 63)	49 (44, 53)	56 (49, 63)	55 (52, 64)	129.5; 0.617	101; 0.255	66.5; 0.733	115; 0.317
STAI-Y2	58 (49, 66)	49 (46, 57)	60 (48, 67)	58 (42, 65)	136.5; 0.796	102.5; 0.079	**119; 0.046**	137.5; 0.822
BDI	15 (5, 24)	8 (5, 15)	17 (7, 25)	17 (8, 27)	124; 0.49	102; 0.082	98.5; 0.307	122.5; 0.458
ASC-12	4 (3, 6)	4 (2, 6)	8 (4, 10)	4 (2, 6)	99; 0.119 ^b^	77; 0.343	**104; 0.013**	178.5; 0.244
MAAS	65 (49, 72)	70 (55, 78)	53 (40, 70)	60 (38, 64)	167.5; 0.438	**25; 0.049**	77; 1.000	178; 0.255

The frequency of headache attacks and medication intake refers to the last three months. Significant results (*p* < 0.05) are reported in bold. *Abbreviations*: TaU + MIND = Mindfulness added to Treatment as Usual group; TaU = Treatment as Usual group. Δ = longitudinal change (T1 - T0); STAI-Y = State-Trait Anxiety Inventory; BDI = Beck Depression Inventory II; ASC-12 = Allodynia Symptoms Checklist; MAAS = Mindful Attention and Awareness Scale; Q1 = first quartile; Q3 = third quartile. Outlier removal was performed in the following cases: a = one outlier (U = 113, *p* = 0.417); b = one outlier (U = 99; *p* = 0.186); c = one outlier (U = 74.5, *p* = 0.028); d = one outlier (U = 119, *p* = 0.552)

### Resting-state fMRI analyses

**Fig. 2:**
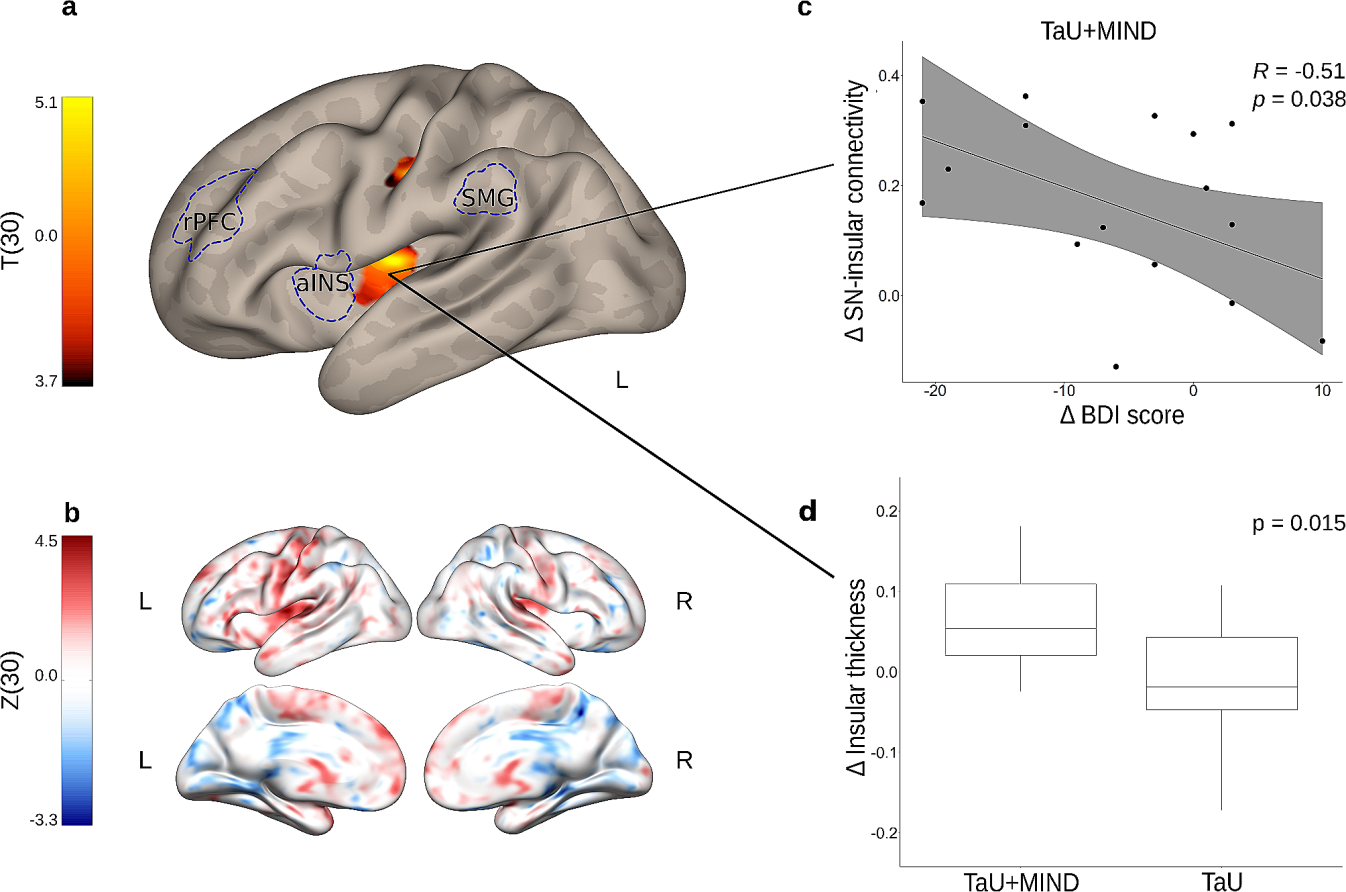
Resting-state fMRI results. Panel (**a**) shows longitudinal differences (Δ = T1 – T0) in SN functional connectivity between TaU + MIND and TaU groups (Δ TaU + MIND vs. Δ TaU). The left sensorimotor cluster is located at MNI −40, -20, +38; the left insular cluster is located at MNI −38, -14, +12. Results are corrected at p-FWE < 0.05 at the cluster level and *p* < 0.001 uncorrected at the voxel level. Three brain regions used as nodes of the SN are reported with dashed lines (rPFC = bilateral rostral prefrontal cortex, aINS = bilateral anterior insula, SMG = bilateral supramarginal gyrus; ACC = anterior cingulate cortex, not shown in the present figure). Panel (**b**) shows the unthresholded longitudinal SN functional connectivity maps **(**Δ TaU + MIND vs. Δ TaU). Panel (**c**) shows a significant negative correlation (error bar = 95% Confidence Interval) between longitudinal functional connectivity changes in SN-left insular cluster and changes in Beck Depression Inventory II (BDI) scores in TaU + MIND group. Panel (**d**) shows longitudinal difference in cortical thickness of the left insular functional cluster in TaU + MIND group compared to the TaU group. *Abbreviations*: *L = left hemisphere; R = right hemisphere; T = t-values(degrees-of-freedom); Z = z-values(degrees-of-freedom)*

At T0, no differences were found between TaU + MIND and TaU groups in seed-based functional connectivity of DMN, SN, and FPN. Longitudinal between-group comparisons (Δ TaU + MIND vs. Δ TaU) showed in TaU + MIND group compared to TaU group increased SN connectivity with a left insular cluster (peak MNI: x=-38, y=-14, z = +12; cluster size = 157 mm^3^; T (30) = 4.94; p-FWE clust.=0.007; p-unc. vox. < 0.0001) and a left sensorimotor cluster (peak MNI: x=-40, y=-20, z = +38; cluster size = 122 mm^3^; T (30) = 5.10; p-FWE clust.=0.026; p-unc. vox. < 0.0001) (Fig. 2a, b). Both clusters showed a large effect size (Cohen’s *d*) (1.72 for left insula, CI 95%=1.08–2.36; 1.99 for left sensorimotor cluster, CI 95%=1.25–2.73). Unthresholded z-maps and significant cluster masks are available at Neurovault (https://identifiers.org/neurovault.collection:14997). Using a probabilistic atlas of the insula [36], we determined that the left insular cluster involved mainly the posterior insula (Anterior Long Gyrus) but also part of the anterior insula (part of Posterior Short Gyrus), while the left sensorimotor cluster was located in the left precentral and postcentral gyri.

No differences were reported between groups in longitudinal seed-based functional connectivity of DMN and FPN.

### Correlational analyses

A significant linear negative correlation between the longitudinal SN-left insular cluster functional connectivity change and the longitudinal variation in Beck Depression Inventory-II scores was reported only in the TaU + MIND group (*r*=-0.51; *p* = 0.038) (Fig. 2c).

### Structural analyses

In the region of interest analysis, the change in cortical thickness extracted from the significant left sensorimotor functional cluster showed no difference in the longitudinal between-group comparison. On the other hand, the longitudinal change in cortical thickness obtained from the left insular functional cluster revealed a significant increase (*p* = 0.015; T (30) = 1.86; Cohen’s *d* = 0.68, indicative of large effect, CI 95%=0.43–0.93, Fig. 2d) in the TaU + MIND group (mean ± standard deviation = 0.047 ± 0.097; range=-0.257-0.181) compared to the TaU group (mean ± standard deviation=-0.012 ± 0.075; range=-0.172-0.108).

**Fig. 3:**
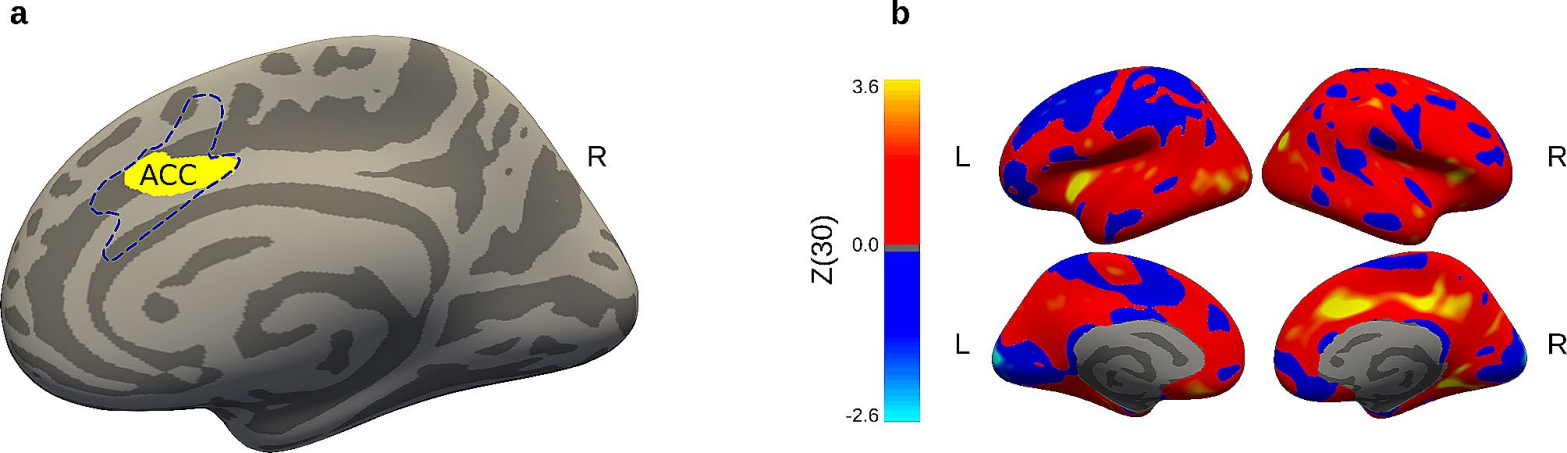
Structural results. Panel (**a**) shows longitudinal between-group vertex-wise differences (Δ TaU + MIND vs. Δ TaU) in right caudal anterior cingulate cortex (ACC) cortical thickness (MNI: x = + 6, y = + 9, z = + 36; p-FWE cluster-wise = 0.02). The ACC brain region used as node of the SN is reported with dashed lines. Panel (**b**) shows the unthresholded longitudinal between-group vertex-wise contrast map (Δ TaU + MIND vs. Δ TaU). *Abbreviations: L = left hemisphere; R = right hemisphere*

In whole-brain vertex-wise analysis, between-group contrast at T0 revealed no difference in cortical thickness. The longitudinal between-group contrast showed a significant increase in the right caudal anterior cingulate cortex cortical thickness (MNI: x = + 6, y = + 9, z = + 36; cluster size = 319 mm^2^; Z (30) = 3.60; p-FWE cluster-wise = 0.02) in the TaU + MIND group compared to TaU group, with an estimated effect size (Cohen’s *d*) of 6.80 (large effect, CI 95%=4.26–9.34) (Fig. 3a, b).

## Discussion

TaU + MIND and TaU patients were largely similar in all demographic and clinical variables, and no neurofunctional differences were observed between groups at T0, confirming their comparability at the baseline. Both groups demonstrated an overall clinical improvement at the follow-up compared to the baseline assessment, indicating the general effectiveness of treatment as usual. Notably, longitudinal between-group comparisons revealed a tendency towards a greater improvement in headache attacks frequency in TaU + MIND compared to TaU patients. Furthermore, longitudinal rs-fMRI between-group comparisons revealed increased SN functional connectivity in the TaU + MIND group with large effect sizes. Specifically, the TaU + MIND group exhibited increased functional connectivity between the SN and a left insular cluster, involving a substantial portion of the posterior insula and part of the anterior insula [36]. Notably, longitudinal functional connectivity changes within the SN-insular cluster were significantly correlated with reduced depression scores in TaU + MIND and associated with an underlying increase of cortical thickness in these patients alone. In addition, increased connectivity was found between the SN and a left sensorimotor cluster. While these results are more prominent in the left hemisphere, unthresholded maps showed a bilateral effect for both insula and sensorimotor clusters. No significant differences were observed in DMN and FPN connectivity. Finally, augmented cortical thickness was observed in the anterior cingulate cortex in vertex-wise analysis with a large effect size.

Since our sample is part of the larger cohort tested by Grazzi et al. [11], our positive clinical findings should be interpreted considering the beneficial effects of the combined TaU + MIND treatment previously reported. With respect to the neuroimaging findings, we observed a central role played by the SN. The SN drives the switching between DMN and FPN [21]. This cyclical mechanism is central to becoming aware of mind-wandering and refocusing attention on the present moment during meditation [37]. The anterior insula, along with the dorsocaudal portion of the anterior cingulate cortex, is a fundamental node of the SN. The increased insular connectivity and cortical thickness observed in the TaU + MIND group likely reflect reinforced within-SN signal organization and neural plasticity, replicating previous findings of SN activity modulation and cortical expansion in both novice and experienced meditation practitioners [12, 16, 38–40]. More specifically, Sezer and colleagues [16] showed that mindfulness practice increases SN connectivity, leading to pain relief and regulation. This is possibly because mindfulness practice encourages nonjudgemental awareness and acceptance (i.e., nonreactivity) of pain sensations [41], and the SN is involved in attention monitoring and emotional and physiological processing of salient stimuli like pain [42, 43]. As mindfulness meditation modulates SN activity, this practice might help reappraise subjective pain experience and detaching from associated negative affective processing, rumination, and catastrophizing. Conversely, connectivity changes in DMN and FPN improve attention control and emotion regulation. On this basis, our findings suggest a significant impact of mindfulness on SN connectivity, possibly associated with changes in pain processing. The distinctive neurobiological profile of the chronic pain patients enrolled in our study may explain their heightened sensitivity to SN functional changes compared to FPN and DMN. This interpretation is also in line with the findings by Michels and colleagues [23] showing increased SN connectivity in CM-MOH patients, suggesting a specific dysregulation of this system. However, we cannot exclude that changes in these two networks might emerge with larger samples as FPN and DMN involvement in mindfulness practice has been observed in recent neuroimaging meta-analyses [14, 16].

Therefore, our study contributes to the growing body of literature linking increased connectivity within the SN to pain processing regulation, supporting the notion that mindfulness-related changes in the SN may influence chronic pain processing [16, 18, 44]. The selective impairment of sensory pain processing in CM-MOH patients has been revealed by diminished activity in insular and cingulate SN nodes in response to noxious mechanical stimulation [24]. Notably, SN activity tends to return to levels similar to those of healthy controls after receiving treatment and medications withdrawal [24]. Nociceptive input is first received and coded in terms of intensity and somatotopic location by the posterior insula [45–47], where a large part of our functional connectivity cluster falls in. Nociceptive information is then transferred to the anterior insula, a key node within the SN, where emotional processing occurs [45–47]. The anterior insula is specifically involved in the integration and recognition of interoceptive information, as well as in pain perception and pain-related emotional reactions [25, 45]. Moreover, this region is a fundamental central autonomic system hub modulating physiological responses to arousing stimuli and regulating internal bodily functions [25]. Furthermore, meta-analytic evidence suggests that dysregulated (re)activity of the anterior insula may represent a robust functional biomarker of chronic pain and a potential target for treatment in chronic pain disorders [26]. Based on this evidence, our findings suggest that mindfulness practice in patients with CM-MOH patients could potentially influence dysregulated pain processing mechanisms by enhancing the functional connectivity within-insula between its posterior and anterior components. This, in turn, could lead to improved emotional processing, regulation of chronic headache-related pain, and autonomic response modulation. On this basis, mindfulness may facilitate a greater conscious acceptance of pain by modulating the activity of brain regions responsible for transforming nociceptive information into the subjective experience of pain.

Additionally, our finding of enhanced SN-insular connectivity in TaU + MIND patients was associated with diminished depression scores. This result is of particular relevance as CM-MOH patients have a greater risk of suffering from depressive disorders with respect to other patients with migraine [48]. Several studies have provided evidence that mindfulness therapy is effective in treating depression symptoms and relapses through improved self-regulation and present-moment embodied awareness [17, 49]. Our result is consistent with the recent Randomized Controlled Trial by van der Velden et al. [50] reporting that mindfulness-related SN connectivity changes were associated with diminished rumination and increased body awareness in depressed patients. Therefore, SN-insular connectivity modulation induced by mindfulness practice may have alleviated depression symptoms in CM-MOH patients by fostering non-judgemental acceptance of painful bodily sensations and attention disengagement from persistent headache-pain-related rumination.

Moreover, in our study, we found additional evidence supporting that mindfulness practice improves bodily pain awareness. Specifically, TaU + MIND patients showed increased connectivity between the SN and the sensorimotor cortex. The anatomical location of our cluster aligns with the somatotopic representation of the face on the lateral part of the sensorimotor cortex [51]. This finding suggests that mindfulness may induce a remodulation of the processing and perception of bodily sensations, including those related to headaches and facial pain in CM-MOH patients, by strengthening the connectivity between brain regions responsible for detecting and selecting internal salient stimuli and those involved in processing sensory information.

Finally, SN connectivity changes observed in our study were also accompanied by cortical thickness neuroplastic expansion in TaU + MIND patients. In particular, the dorsocaudal anterior cingulate cortex cluster observed in the longitudinal vertex-wise analysis was largely included within the cingulate node of the SN. Meta-analytic studies have shown that mindfulness meditation influences both dorsocaudal cingulate anterior cortex activity and structure [12, 39]. Notably, Zeidan and colleagues [18] reported that experimentally-induced-pain intensity was associated with activations in this region and anterior insula. Interestingly, these regions were active both in meditation and experimental pain tasks [18], suggesting a potential substrate for mindfulness-related pain modulation. Moreover, the dorsocaudal anterior cingulate cortex has multifaceted functions, including cognitive control, conflict monitoring [17, 52], central autonomic system regulation, and integration of negative affect with cognition [25]. On this ground, the cingulate cortex expansion that we observed in the TaU + MIND group may lead to improved cognitive processing of emotional and nociceptive information facilitating non-judgmental acceptance and top-down regulation of headache-related pain [53].

Our findings should be considered in light of some relevant limitations. The occurrence of COVID-19 pandemic considerably interfered with our Trial. Unfortunately, this rendered impossible for several patients to undergo the follow-up assessment at our institute, thus reducing our sample size. Therefore, our findings should be interpreted with great caution and regarded as exploratory and preliminary. While we observed large effect sizes and the within-subject comparison design increases statistical power and reliability [54], further confirmatory studies with larger samples are necessary to validate our findings. This is particularly true since the reproducibility of rs-fMRI findings in the context of migraine is still a matter of debate, in particular because of mixed evidence from studies with limited samples [55]. The restricted sample size also hindered our possibility to examine the influence of medication overuse profiles and prophylaxis on functional connectivity. Moreover, our results are partially limited in their generalizability because the study was performed in a single institute, a third-level headache centre, and the patients who attended the structured withdrawal had a very severe clinical profile. A further limitation consists in the lack of data regarding therapy adherence between the two timepoints. Although we included a question during follow-up visits to assess adherence to the 7–10 min daily self-practice, in line with Grazzi et al. [11], we chose not to integrate this data into our analysis for several reasons: recall bias linked to the assessment timing; the disruption of patients’ daily routines due to the COVID-19 pandemic; and the relatively lower reliability of this method compared to contemporary techniques, such as mobile device applications.

Despite these limitations, our research represents the first longitudinal study providing a description of the neurofunctional correlates of the beneficial effects of mindfulness practice in CM-MOH patients. Overall, the structural and functional changes observed in cingulate and insular SN regions in the TaU + MIND group may be linked to the mindfulness-induced remodulation of the significance attributed to salient sensory events. Mindfulness practice can regulate the autonomic activity by modulating both the physiological response to bodily salient stimuli, and the emotional and cognitive processing of nociceptive information. Chronic headache patients may then feel both more aware and capable of distancing themselves from their bodily sensations, thus accepting the experience of headache pain more consciously.

In this context, it has been suggested that CM-MOH might be characterized by both lifelong-stable brain modifications that predispose one individual to medication overuse (i.e., “trait-like” neuromechanisms) and transient brain adaptations (i.e., “state-like” neuromechanisms) that can be reversed by medication discontinuation [22]. Our results suggest that protracted mindfulness practice may impact state-like reversible neural modifications, inducing a remodulation of the neurofunctional organization of pain-processing brain regions that are dysregulated in CM-MOH patients.

## Conclusions

The present study reported that mindfulness practice added to treatment as usual in CM-MOH patients specifically increased SN functional connectivity and cortical thickness, with an associated clinical improvement. Our results suggest that the known beneficial effects of mindfulness practice are underpinned by SN neurofunctional changes potentially achieved through the modulation of physiological, emotional, and cognitive aspects of nociceptive information processing. Ultimately, this process may enhance body awareness and improve the ability to accept and cope with the subjective experience of pain. These exploratory findings contribute to the growing understanding of how mindfulness practice may positively impact brain functioning, emotional regulation, and pain management in patients with CM-MOH, encouraging widespread research on this non-pharmacological intervention for chronic headache management.

## Data Availability

Unthresholded z-maps and significant cluster masks are available at Neurovault (https://identifiers.org/neurovault.collection:14997). Other data may be made available upon reasonable request.

## Abbreviations

CM-MOH: Chronic Migraine associated with Medication Overuse Headache
TaU: Treatment as Usual
TaU + MIND: Mindfulness added to Treatment as Usual
DMN: Default Mode Network
SN: Salience Network
FPN: Fronto-Parietal Network
rs-fMRI: resting-state fMRI
T0: Baseline
T1: Follow-up
Δ: Longitudinal difference (Follow-up – Baseline)
